# Reassortment and distinct evolutionary dynamics of Rift Valley Fever virus genomic segments

**DOI:** 10.1038/srep11353

**Published:** 2015-06-23

**Authors:** Caio C. M. Freire, Atila Iamarino, Peinda O. Ly Soumaré, Ousmane Faye, Amadou A. Sall, Paolo M. A. Zanotto

**Affiliations:** 1 Laboratory of Molecular Evolution and Bioinformatics, Department of Microbiology, Biomedical Sciences Institute, University of Sao Paulo, Sao Paulo, Brazil; 2 Institut Pasteur de Dakar, Dakar, Senegal

## Abstract

Rift Valley Fever virus (RVFV) is a member of Bunyaviridae family that causes a febrile disease affecting mainly ruminants and occasionally humans in Africa, with symptoms that range from mid to severe. RVFV has a tri-segmented ssRNA genome that permits reassortment and could generate more virulent strains. In this study, we reveal the importance of reassortment for RVFV evolution using viral gene genealogy inference and phylodynamics. We uncovered seven events of reassortment that originated RVFV lineages with discordant origins among segments. Moreover, we also found that despite similar selection regimens, the three segments have distinct evolutionary dynamics; the longer segment L evolves at a significant lower rate. Episodes of discordance between population size estimates per segment also coincided with reassortment dating. Our results show that RVFV segments are decoupled enough to have distinct demographic histories and to evolve under different molecular rates.

Rift Valley Fever (RVF) is an emergent arthropod-borne illness that primarily affects ruminants in eastern and sub-Saharan Africa^1^,^2^, causing severe socioeconomic impact in both animals and humans^3^. The causative agent of this disease is a Phlebovirus of the Bunyaviridae family, the RVF virus (RVFV). Since RVFV may be successfully vectored and widely spread by more than 30 distinct mosquito species^4^,^5^, it has become a major public health concern not only in Africa^6^, as demonstrated by the recent outbreaks in Saudi Arabia and Yemen^7^. The virus spread can be scattered and erratic, but fast and efficient^8^ and can cause high mortality in humans and animals, with rates around 30%, as was the case during the Kenyan outbreak in 2007^9^.

Like all Bunyaviruses, RVFV has a three-segmented RNA genome organized in three segments: L (large), M (medium) and S (small). The L and M segments encode in the complementary sense, while the S segment has an ambisense arrangement. The L segment encodes the RNA-dependent RNA polymerase^10^, the M segment codes for the envelope glycoproteins G1 and G2 and two non-structural proteins with 14 and 78 kDa^11^. The S segment encodes the nucleocapsid protein (N) on the complementary sense, and a non-structural protein (NSs) in the other^12^ that plays a major role in innate immunity and interacts with interferon signaling pathways^13^.

This segmented genome permits natural reassortment^14^,^15^,^16^, an event that requires that distinct viral strains co-infect a single cell at the same time, and may increase viral genetic diversity^17^. RVFV is able to infect an extensive plethora of vertebrate hosts (lambs, goats, cattle, rodents and humans) and arthropod-vectors^6^, which may help to maintain the virus at a given place in space and facilitates co-infection in time. This begs the question of the importance of reassortment during the recent dissemination of the virus. A question that can be investigated, given that RVFV genome organization permits unraveling distinct evolutionary histories among segments reconstructed using the coalescent theory^18^. Although already observed in nature, estimates of reassortment in RVFV are made difficult by the scarcity of complete genomic sequences from several localities, such as West Africa. Moreover, analyses done on concatenated or combined datasets, using more than one segment in the same genealogy, did not account for reassortment as a potential source of systematic error during phylogenetic inference^19^. We studied reassortment in RVFV using a framework developed initially for influenza virus^20^. We also investigated whether dynamics of viral genomic segments could have incongruous (*i.e.*, unlinked) evolutionary histories due to reassortment^21^.

## Results and Discussion

### Adjacency patterns in phylogenies show reassortment events

**Figure 1:**
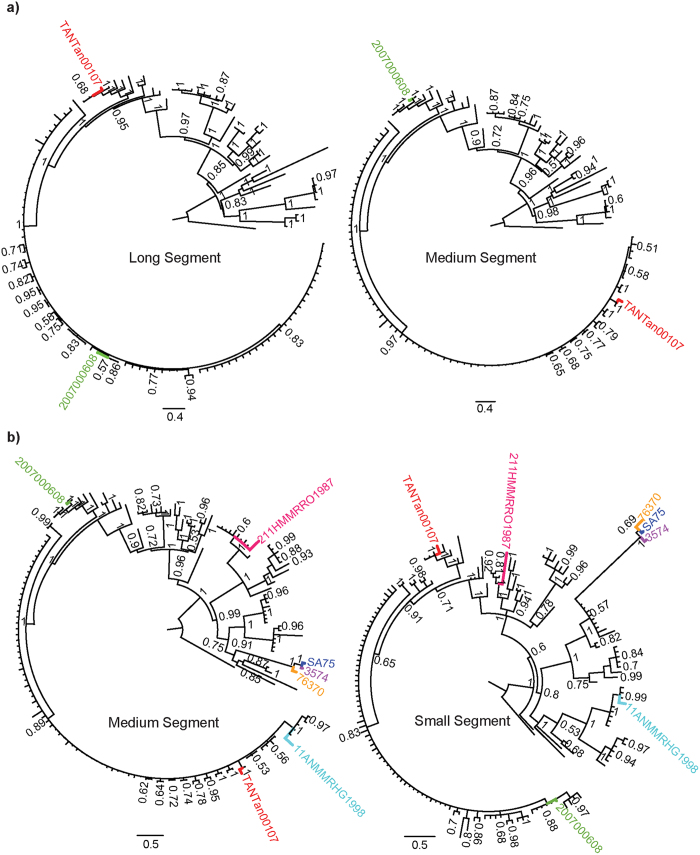
Reassortments revealed by the incongruities among phylogenies for RVFV segments. **a)** Reassortments between L and M segments. **b)** Reassortments between M and S segments. Pairs of reassortant taxa are show in colors specific to the reassortant pair. The order of taxa is the same between each pair of trees. Parental strains can be observed in Figure S1. Only reassortant taxon names are shown for clarity. Reconciled, parallel trees, showing reassortants, including all taxon names are shown in Figure S1.

We analyzed the posterior set of trees (PST) generated during independent phylodynamic Monte Carlo Markov Chain (MCMC) runs for all segments and identified two RVFV lineages with discordant phylogenetic histories for M and for L and S segments. Our results confirmed previous findings that the Kenyan bovine isolate 2007000608 was formed via reassortment of M segments^22^ (Table 1 and green taxon labels in Fig. 1). Furthermore, we observed a new reassortment event in the Tanzanian lineage TANTan00107 (Table 1 and red taxon labels in Fig. 1), sampled from a human source. Since TANTan00107 was used in concatenated datasets^19^, this finding demonstrates the importance of checking genome sequences with recombination and reassortment detection programs such as RDP4^23^ and GiRaF^20^, respectively, to prevent systematic errors during phylogenetic inferences. Moreover, we investigated the occurrence of reassortment between pairs of segments (L and M, L and S, M and S), using the largest datasets (Fig. 1 and Table 1). We found no additional reassortants between the L and the other two segments. However, we were able to detect five additional new reassortants between M and S segments (Fig. 1B and Table 1). Three of these reassortant lineages (76370, SA75 and 3574), isolated in Zimbabwe and South Africa in human, cattle and sheep (Table 1), form a highly supported reassortant cluster that reappears in independent phylogenies for both the M and S segments (orange, purple and blue taxon labels in Fig. 1B). The other two reassortants were from Mauritania (211HMMRRO1987 and 11ANMMRHG1998, pink and cyan taxon labels in Fig. 1B), and were isolated from human and sheep, respectively (Table 1). The parental relationships can be accessed in Figure S1.

### The genetic diversity inferred by patristic distances indicates distinct time scales for each segment

Since a segmented genome allows separate evolutionary histories per segment^18^,^21^, we investigated the amount of divergence for each segment by estimating patristic distances across viral gene genealogies, which may vary more than genetic distances and thus better capture discordances^24^. Pairwise distance distributions for the three datasets (for segments L, M and S with 120 sequences each) deviated from normality and were significantly different (χ^2^ = 136.21 d.f. = 2 and *p*-value < 2.2E-16 in Kruskal-Wallis test) after an exhaustive comparison among segments. Crucially, we obtained the ‘difference values’ of 689.73, 513.84 and 1203.57 for comparisons between L and M, L and S, and M and S, respectively. All values were greater than the critical value of 247.76, for the significance level (α) = 0.05. Nevertheless, we found no evidences of recombination intra-segment, which corroborates the notion that the segments have different amounts of genetic changes, indicating distinct time-scales for the segments.

### Similar selection regimens for each segment support reassortment as the main driver of individual segment evolution

**Figure 2:**
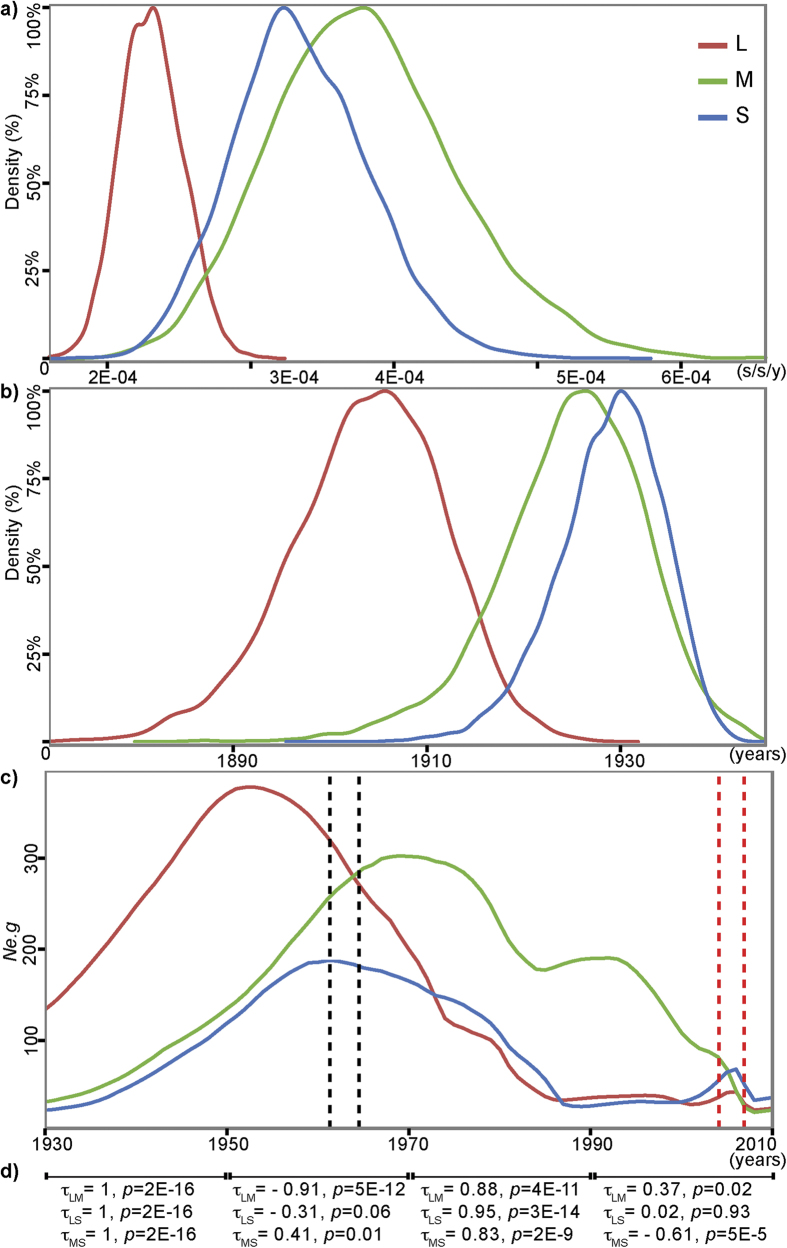
Phylodynamic estimates for the best-fit evolutionary rate for each segment. **a)** Distribution of evolutionary rates (μ) estimated during MCMC convergence. **b)** Distribution of estimated the root (TMRCA) for each segment. **c)** BSP showing the effective population size (*Ne.g*) estimated for L, M and S segments. The vertical colored intervals indicate the estimates of the node age of detected reassortant lineages (red for 2007000608 and TANTan00107; gray for SA75, 3574 and 76370). The node ages are shown in Figure S2. **d**) Correlation between pairs of *Ne.g* for 20 years intervals. Kendall correlation coefficients (τ) < 0 indicated anti-correlation in the intervals and *p*-values < 0.05 point to statistical significance of the correlations. All estimates are based on datasets with 120 sequences.

Differences in selection regimen among segments could explain the observed differences among patristic distances. Therefore, we investigated selection acting at the codon level on each segment. We only found sites with statistical significance under purifying selection for all analyzed genes (ω < 0 and *p*-value < 0.05, in Table S1). We compared with Fisher’s exact test the amount of negatively selected codons with statistical significance and found 98 codons out of 2066, 58 out of 1197, and 20 out of 510 for L, M and S respectively. However, there was no significant difference among the proportions of codons under selection for each segment (*p*-value = 0.74). Moreover, we compared the intensity of selection on each segment with ω, using the Kruskal-Wallis test and found no significant differences among ω for each segment (χ^2^ = 3.92, d.f. = 2 and *p*-value = 0.14). Therefore, our results did not support apparent differences in the selection regime acting on codons of each segment. Additionally, we estimated the Tajima’s D for each segment and found that they were significantly negative and similar for all segments (L: D = −3.19, *p*-value = 0.0013; M: D = −3.18, *p*-value = 0.0014; S: D = −3.32, *p*-value = 0.0009). These similar negative Tajima’s D values did not change after excluding reassortant taxa out of the analysis, and agree with our findings on purifying selection. Negative Tajima’s Ds also point to possible past bottlenecks, also corroborated by the drop in effective population size (*Ne.g*) after 1970 for all segments (Fig. 2C). Nevertheless, since the selective regimen is similar among segments and, given the significant differences in patristic distance distributions, these results strongly suggest that RVFV segments are evolving under different time-scales, which constitutes an independent source of evidence for reassortment.

### RVFV segments have different evolutionary dynamics that relates to reassortment

In principle, reassortment events could decouple evolutionary dynamics for different genomic segments^18^,^21^. Therefore, we estimated the evolutionary rates (μ) for each segment independently and compared the likelihoods using the Akaike’s information criterion through Markov chain Monte Carlo (AICM) and path-sampling tests to choose the μ that better fit our data (Table S2). The best μ, obtained from BEAST, for the L segment was 2.31E-4 substitutions per site per year (s/s/y) ranging from 1.88E-4 to 2.72E-4 (within 95% highest posterior densities, HPD) (Fig. 2A). For the M segment, the mean μ of 3.80E-4 s/s/y (HPD = 2.53E-4 to 5.05E-4 s/s/y) had a better fit for the data. Finally, the S segment had a μ of 3.36E-4 s/s/y (HPD = 2.42E-4 to 4.26E-4 s/s/y). In sum, the mean rate for M segment was at least 1.65 times higher than μ for L and 1.45 times that for S. These differences implied in an older root estimate for the L segment (Fig. 2B) and overlapping roots for S and M. Importantly, the mean effective population size (*Ne.g*) of each segment experienced detectable periods of anti-correlation (Fig. 2D), which had significant negative Kendall coefficients (τ < 0 and *p*-value ≤ 0.05 in the Fig. 2D) in two time intervals: (*i*) between 1930 to 1950 and (*ii*) 1990 and 2010. Interestingly, these two periods of anti-correlation superimpose the estimated ages for the coalescence of reassortant lineages (Figure S2 and vertical intervals in Fig. 2C). Although the nature of the association between the anti-correlation in the BSP with the estimated times for the coalescence nodes of reassortant lineages remains unclear, it could also suggest decoupling of the evolutionary dynamics among RVFV segments. Nevertheless, our findings support the notion that reassortment events in both the S and M segments took place during the recent evolutionary past of RVFV (Fig. 1B and S1B). This could imply some form of linkage between these two genes, which is consistent with the fact that S and M interact during viral morphogenesis^25^. Remarkably, we found reassortant lineages amongst human samples (Table 1), a concerning result given that reassortment events in Bunyaviruses are known to have originated pathogenic new lineages from milder parental ones^26^.

## Methods

### Sequence datasets

We used our previously generated sequences^8^ and all other RVFV sequences available in GenBank (http://www.ncbi.nlm.nih.gov/genbank/). Only sequences with available date of isolation were added in our dataset (Table S3). Sequences were aligned using the multiple alignment program Clustal Omega^27^ and curated with SeaView v4.4.0^28^. To prevent potential biases during phylogenetic inference due to recombination, we first analyzed all sequences from each segment with RDP4 program that incorporates RDP, GENECONV, Chimaera, Maxchi, Bootscan, SiScan and 3Seq methods^23^. Only recombination events with *p*-values ≤0.05 that were detected by three or more methods were considered, employing the Bonferroni correction to avoid false positive results. We then selected the RVFV lineages with available sequences for the three segments (L, M and S), which resulted in three datasets with 120 sequences each. We also selected the lineages with pairs of segments available in all combinations (L/M, L/S, and M/S), resulting in datasets with 127, 128 and 162 sequences, respectively.

### Reassortment detection analyses

To generate a posterior set of trees (PST) we used a Markov Chain Monte Carlo (MCMC) approach with MrBayes v3.2.1^29^, employing a general time reversible substitution model (GTR)^30^ with a gamma distributed rate variation (Γ)^31^ and a proportion of invariable sites (I). MCMC stationarity of two independent runs of 4 chains each (3 heated and 1 cold) was obtained after 20 millions generations, and trees were sampled every 2000. Reassortment events were inferred using GiRaF v1.02 program, which compares a distribution of phylogenetic trees of different segments searching for groups of discordant splits of the trees using a biclique algorithm^20^. We used the PST for GiRaF searches, discarding the first 500 trees, culling splits with frequencies less than 5%, and set a confidence threshold of 70% to report a reassortment event.

### Patristic distances analyses

Firstly, 100 maximum likelihood (ML) phylogenetic trees were independently inferred using GARLI v2.0^32^, that uses a stochastic algorithm to estimate simultaneously the best topology, branch lengths and substitution model parameters that maximize the log Likelihood (lnL). Assuming that simpler models would be satisfactorily accounted for when estimating transition probabilities with the GTR+Γ+I model, we selected the tree with best lnL estimated with it. The patristic distances were calculated from the best tree for each segment with the R-package adephylo 1.16^33^. The differences among patristic distances were accessed with Kruskal-Wallis rank sum test from R-program (http://www.r-project.org/). We evaluated the differences among patristic distances, using the multiple comparison test after Kruskal-Wallis from the R-package pgirmess v1.5.9^34^.

### Selection analyses

To evaluate selection patterns on the coding sequences for each segment: polymerase in the L segment, glycoprotein in M, nonstructural and nucleocapsid in S; we estimated the difference (ω = *d*N-*d*S) between the non-synonymous (*d*N) and synonymous (*d*S) rates per codon sites, using the single likelihood ancestor counting (SLAC) algorithm with HyPhy v2.11^35^, assuming α = 0.05. In this way, ω greater than zero suggests directional selection, while values below zero indicate purifying selection. We used the Fisher’s exact test to investigate the differences in the total number of sites under selection among the segments and Kruskal-Wallis rank sum test to access the differences among ω for each segment. Both tests were done with the R program. Moreover, to evaluate neutral patterns of evolution, Tajima’s D were estimated for L, M and S datasets; using the R-package pegas v0.5-1^36^.

### Phylodynamics

Since we had dates of isolation for each RVFV sequence, we estimated coarse substitution rates per site per year (μ) using Path-O-Gen v1.4 (http://tree.bio.ed.ac.uk/software/pathogen/) with the best ML trees for each segment to use as priors during Bayesian inferences. In addition, Maximum Clade Credibility (MCC) trees were inferred using a MCMC approach under GTR+Γ+I and a relaxed (uncorrelated lognormal) molecular clock^37^ using program BEAST v1.8.0^38^, with the previously estimated μ values as priors. Moreover, we used the variation in effective population size (*Ne.g*) to infer the viral demography with Bayesian Skyride Plots (BSP)^39^. MCMC convergence was obtained for four independent runs with 100 million steps, which were sufficient to obtain a convergence, as inspected by effective sample sizes (ESS) values above 200 for all parameters. Since we obtained different rates for each segment, we re-ran BEAST changing the mean rate among segments (Table S2). To test the model that best-fit the data we employed a Akaike’s information criterion through Markov chain Monte Carlo (AICM) test^40^, implemented in Tracer v1.6 (http://tree.bio.ed.ac.uk/software/tracer/). Also, the suitability of the μ prior to the data was tested again with path-sampling (PS) and stepping-stone (SS) algorithms^40^. Because our Bayesian Skyride were evidently not stationary at the time span of the observations, we evaluated the correlation between pairs of *Ne.g* for segments using Kendall τ correlation coefficient, to test for the correlation in intervals of 20 years between 1930 and 2010. To access the significance of correlations, we adopted α = 0.05.

## Additional Information

**How to cite this article**: Freire, C. C. M. *et al.* Reassortment and distinct evolutionary dynamics of Rift Valley Fever virus genomic segments. *Sci. Rep.* **5**, 11353; doi: 10.1038/srep11353 (2015).

## Supplementary Material

Supplementary Information

## Figures and Tables

**Table 1:** RVFV reassortant lineages.

Strain	Country	Year	Host	L accession number	M accession number	S accession number	Reference
2007000608	Kenya	2007	Cattle	EU574023	EU574049	EU574079	22
211HMMRRO1987	Mauritania	1987	Human	Unavailable	JN995301	JN995253	8
11ANMMRHG1998	Mauritania	1998	Sheep	Unavailable	JN995312	JN995264	8
SA75	South Africa	1975	Human	DQ375428	DQ380189	DQ380175	16
3574	South Africa	1974	Sheep	JF784386	JF784387	JF784388	41
TANTan00107	Tanzania	2007	Human	HM586959	HM586970	HM586981	19
76370	Zimbabwe	1970	Cattle	DQ375426	DQ380188	DQ380165	16
