# Supplementary material for: Reassortment and distinct evolutionary dynamics of Rift Valley Fever virus genomic segments
Source: Sci Rep. 2015 Jun 23;5:11353. doi: 10.1038/srep11353 (PMC4477411; doi:10.1038/srep11353)
Supplement: Supplementary Information [file srep11353-s1.pdf]

Supporting information

Reassortment and distinct evolutionary dynamics of Rift Valley Fever virus genomic segments

Caio C. M. Freire[1], Atila Iamarino[1], Peinda O. Ly Soumaré[2], Ousmane Faye[2], Amadou A. Sall[2], Paolo M. A. Zanotto[1]*

1. Laboratory of Molecular Evolution and Bioinformatics, Department of Microbiology, Biomedical Sciences Institute, University of Sao Paulo, Sao Paulo, Brazil.
2. Institut Pasteur de Dakar, Dakar, Senegal.

*Corresponding author: Paolo M. A. Zanotto. Laboratory of Molecular Evolution and Bioinformatics, Department of Microbiology, Biomedical Sciences Institute – ICBII, University of São Paulo, Av. Prof. Lineu Prestes, 1734 São Paulo - SP - Brazil – 05508- 000. E-mail: pzanotto@usp.br. Phone: +55 1130918453. Fax: +55 1130917354.

a)

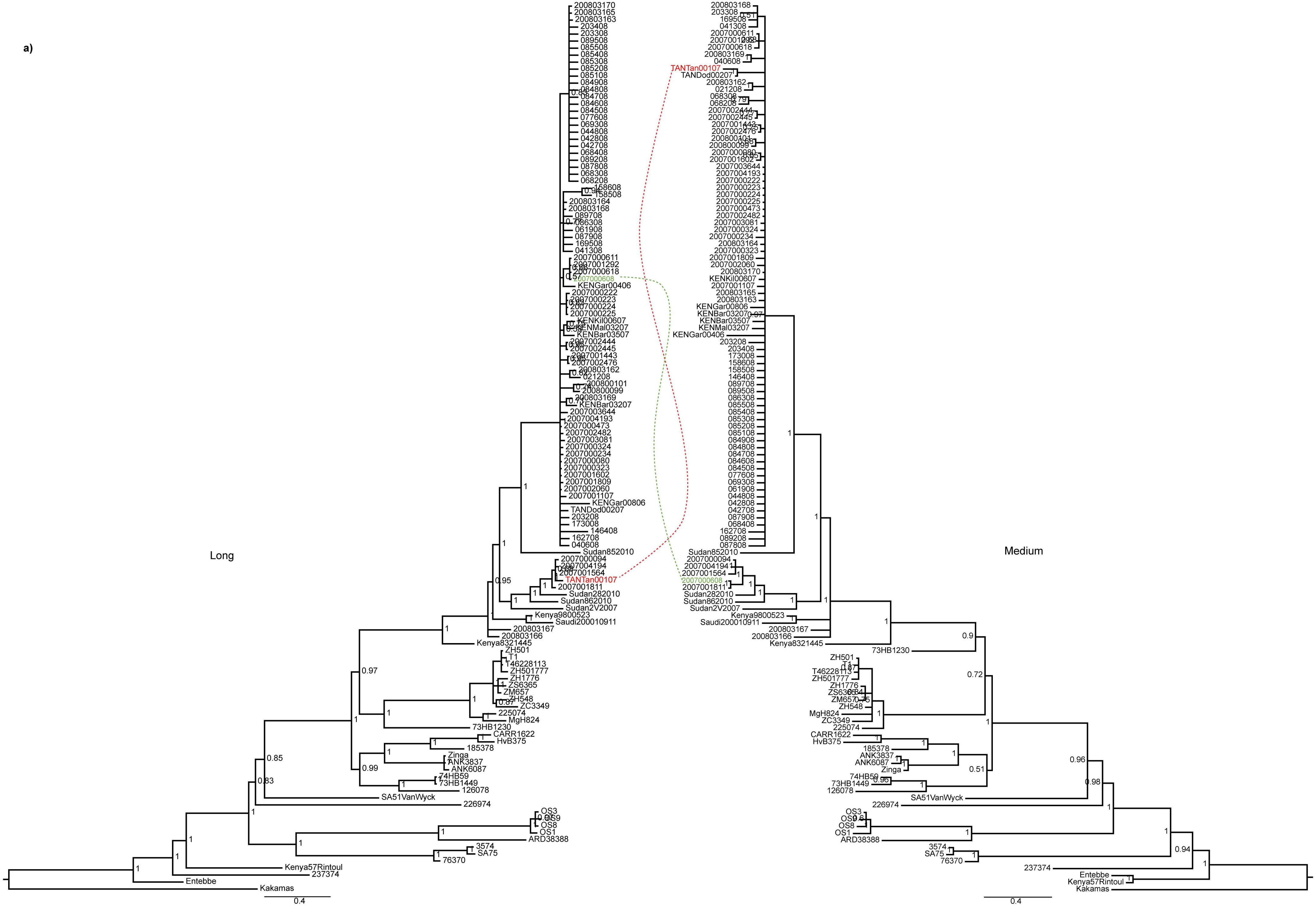

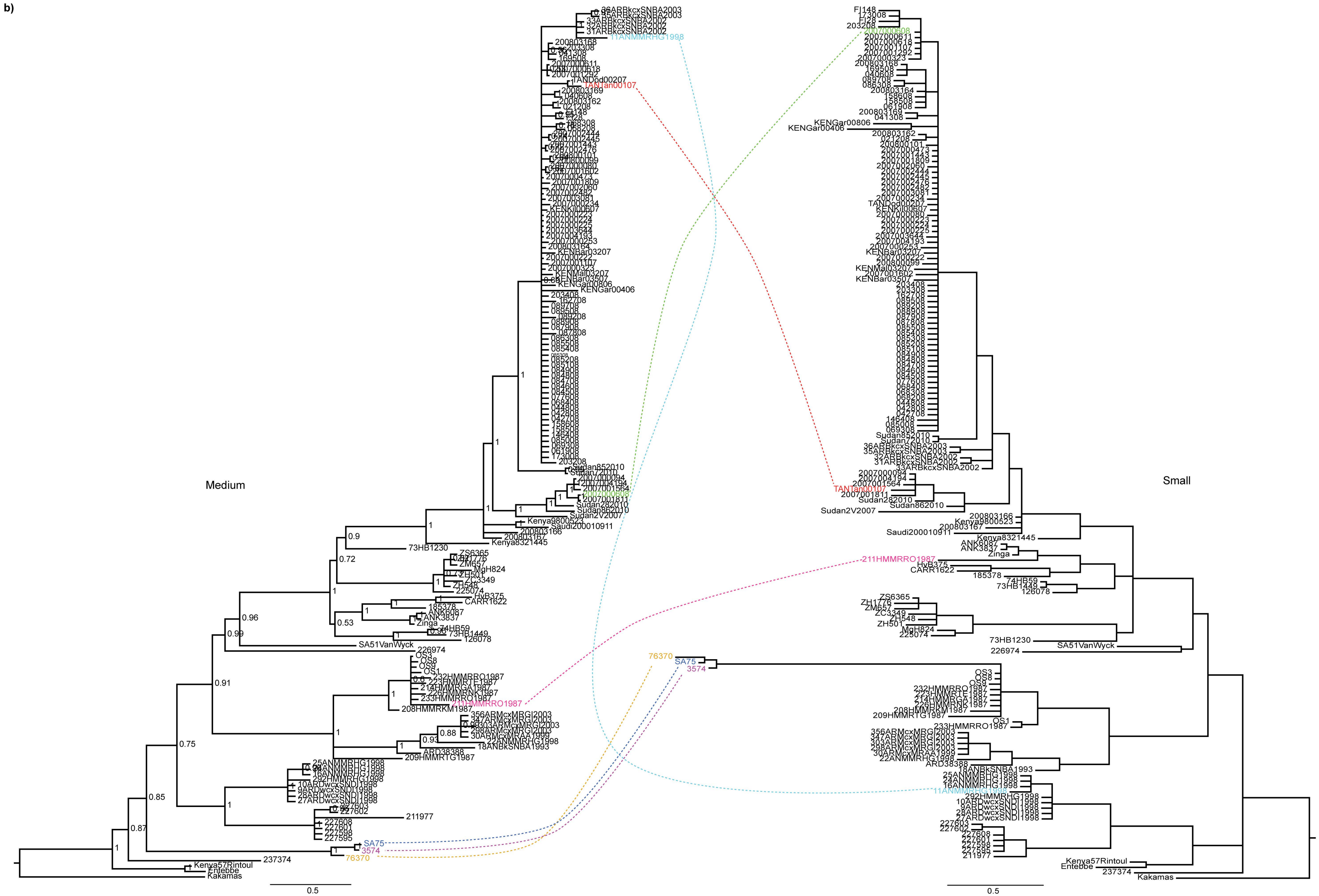

Figure S1. Paired phylogenetic trees for RVFV segments showing phylogenetic discrepancies among reassortant lineages. A – Phylogenetic trees for L and M. B – Phylogenetic trees for M and S. Posterior probability support values are shown close to the nodes. Pairs of reassortants are colored accordingly.

a)

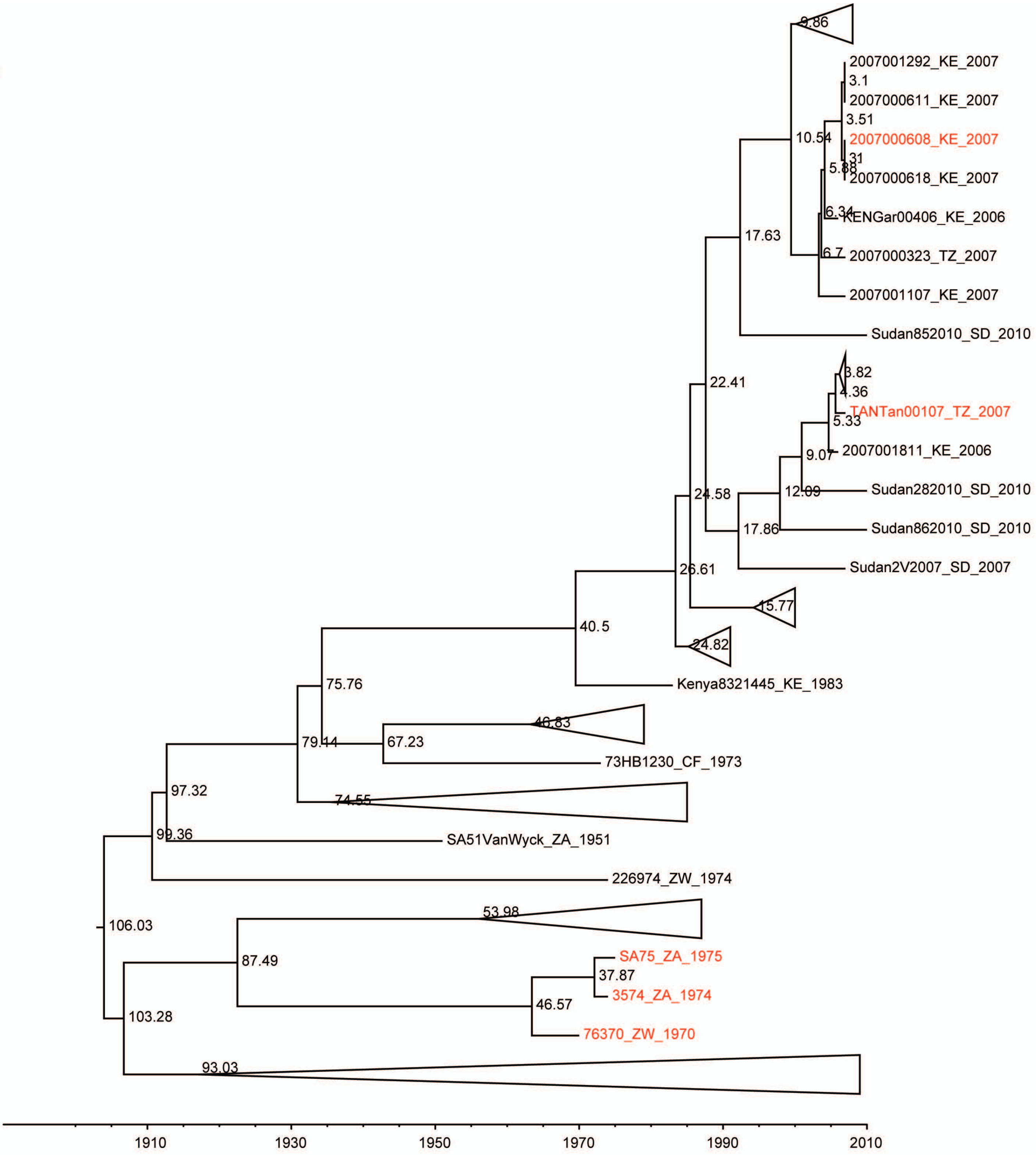
9.86
2007001292_KE_2007
3.1
2007000611_KE_2007
3.51
10.54
2007000608_KE_2007
31
5.88
2007000618_KE_2007
6.34
KENGar00406_KE_2006
17.63
6.7
2007000323_TZ_2007
2007001107_KE_2007
Sudan852010_SD_2010
3.82
22.41
4.36
TANTan00107_TZ_2007
5.33
9.07
2007001811_KE_2006
24.58
12.09
Sudan282010_SD_2010
17.86
Sudan862010_SD_2010
26.61
Sudan2V2007_SD_2007
15.77
40.5
24.82
75.76
Kenya8321445_KE_1983
46.83
79.14
67.23
73HB1230_CF_1973
97.32
74.55
99.36
SA51VanWyck_ZA_1951
226974_ZW_1974
106.03
53.98
87.49
SA75_ZA_1975
37.87
3574_ZA_1974
103.28
46.57
76370_ZW_1970
93.03
1910
1930
1950
1970
1990
2010

b)

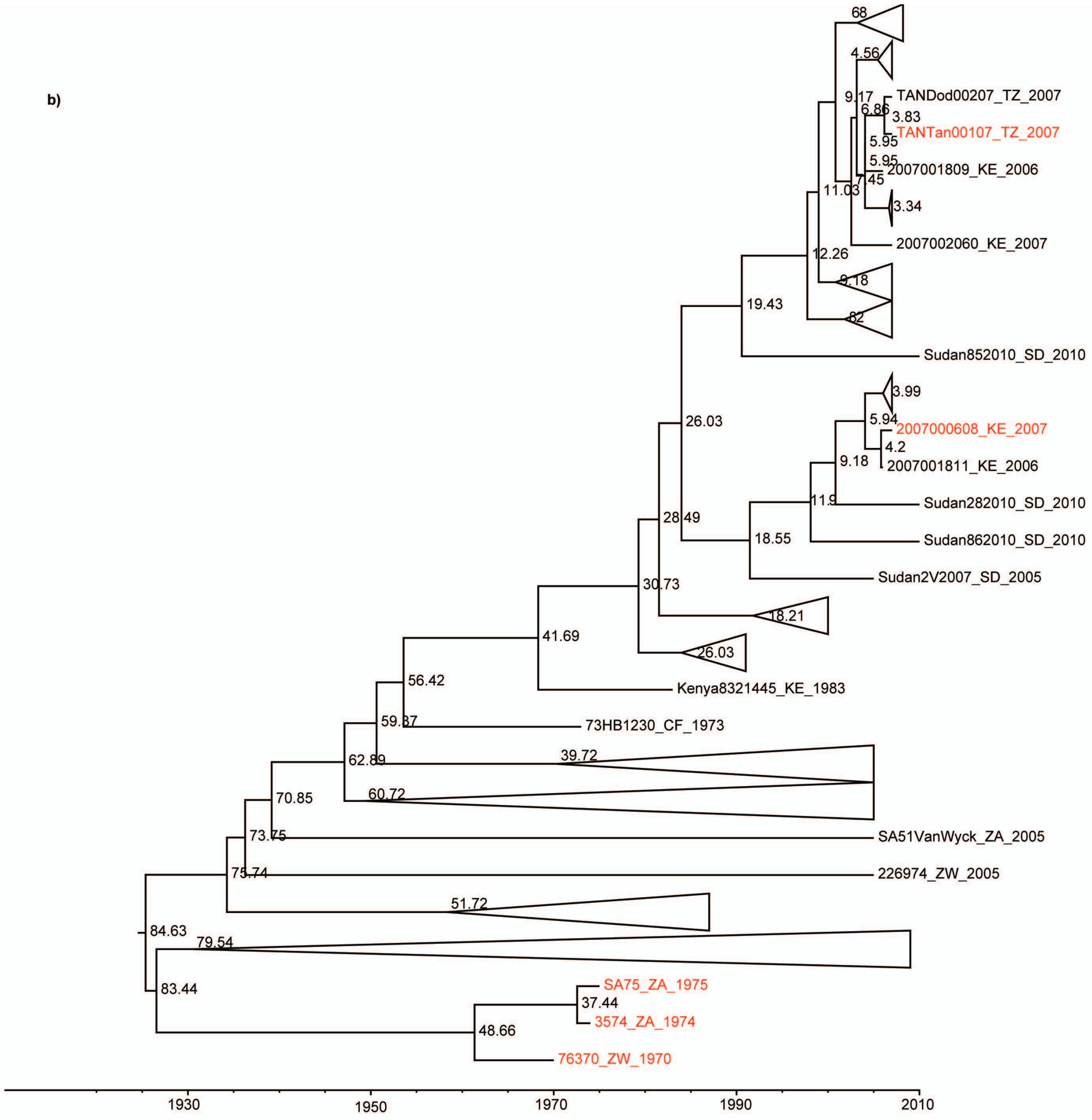
68
4.56
9.17
6.86
TANDod00207_TZ_2007
3.83
TANTan00107_TZ_2007
5.95
5.95
2007001809_KE_2006
7.45
11.03
3.34
2007002060_KE_2007
12.26
9.18
82
19.43
Sudan852010_SD_2010
3.99
26.03
5.94
2007000608_KE_2007
4.2
9.18
2007001811_KE_2006
11.9
Sudan282010_SD_2010
28.49
18.55
Sudan862010_SD_2010
Sudan2V2007_SD_2005
30.73
18.21
41.69
26.03
56.42
Kenya8321445_KE_1983
59.37
73HB1230_CF_1973
62.89
39.72
70.85
60.72
73.75
SA51VanWyck_ZA_2005
75.74
226974_ZW_2005
51.72
84.63
79.54
83.44
SA75_ZA_1975
37.44
3574_ZA_1974
48.66
76370_ZW_1970
1930
1950
1970
1990
2010

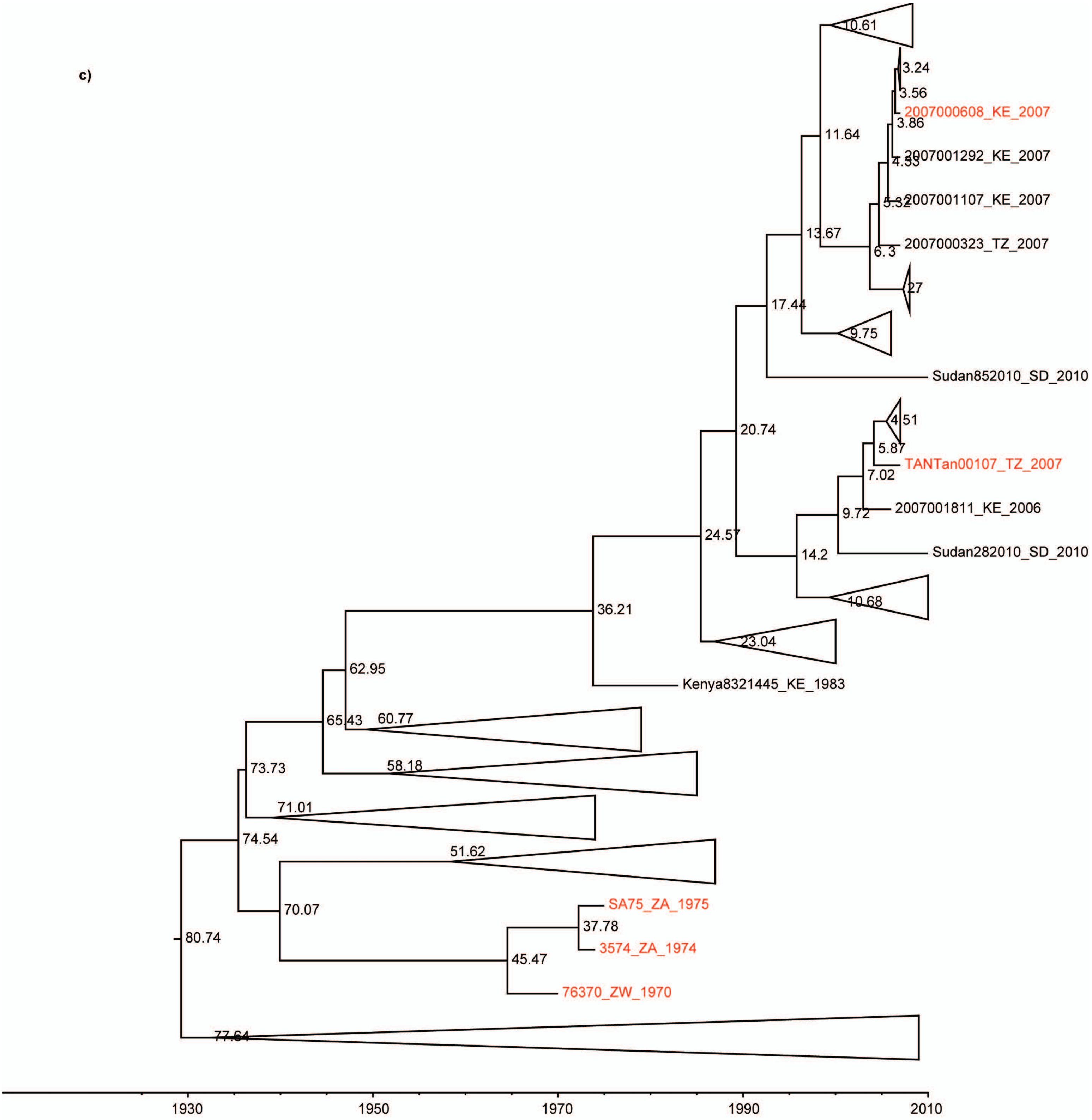

Figure S2. Time-scaled MCC trees, showing times of reassortment. A – MCC tree for L segment. B - MCC tree for M segment. C- MCC tree for S segment. Labels of reassortants are colored in red. TMRCAs are shown close to the nodes.

Table S1. Significant detected codon sites under purifying selection in RVFV.

Aa position	dN-dS	p-value	Gene
27	-11.6951	0.02923	Polymerase
47	-3.36074	0.02841	Polymerase
77	-3.36074	0.02841	Polymerase
90	-3.0764	0.03704	Polymerase
105	-3.0764	0.03842	Polymerase
119	-4.10187	0.01235	Polymerase
146	-4.10187	0.01235	Polymerase
165	-3.22378	0.03365	Polymerase
168	-4.97607	0.00478	Polymerase
178	-3.36074	0.02841	Polymerase
201	-3.0764	0.03704	Polymerase
230	-3.36074	0.02841	Polymerase
253	-3.0764	0.03704	Polymerase
307	-3.36074	0.03096	Polymerase
308	-3.36074	0.02841	Polymerase
337	-4.10187	0.01235	Polymerase
355	-4.47188	0.00921	Polymerase
401	-5.60124	0.00265	Polymerase
416	-3.0764	0.03704	Polymerase
472	-3.201	0.03288	Polymerase
484	-10.353	0.03302	Polymerase
502	-3.0764	0.03704	Polymerase
533	-4.48099	0.00867	Polymerase
536	-3.0764	0.03704	Polymerase
538	-3.36074	0.02841	Polymerase
572	-3.36074	0.03096	Polymerase
588	-3.34039	0.0301	Polymerase
612	-3.0764	0.0373	Polymerase
697	-3.36074	0.02841	Polymerase
722	-3.36074	0.02841	Polymerase
745	-4.10187	0.01325	Polymerase
793	-5.12734	0.00412	Polymerase
799	-3.36074	0.02841	Polymerase
805	-3.36074	0.02841	Polymerase
834	-5.60124	0.00265	Polymerase
869	-3.0764	0.03704	Polymerase
885	-3.36074	0.02975	Polymerase
887	-3.36074	0.02841	Polymerase
930	-4.48099	0.00867	Polymerase
933	-3.36074	0.02975	Polymerase
961	-4.10187	0.01235	Polymerase
989	-3.35158	0.03	Polymerase
1004	-3.3509	0.03002	Polymerase
1065	-6.72149	0.00096	Polymerase
1077	-3.36074	0.03096	Polymerase
1084	-3.35217	0.02998	Polymerase
1126	-3.36074	0.02975	Polymerase
1129	-3.0764	0.03704	Polymerase
1135	-4.09127	0.01338	Polymerase
1177	-4.268	0.01053	Polymerase
1206	-3.0764	0.03704	Polymerase
1209	-3.0764	0.03723	Polymerase
1261	-4.48099	0.00867	Polymerase
1269	-3.0764	0.03704	Polymerase
1270	-3.36074	0.02841	Polymerase
1276	-3.35449	0.02992	Polymerase

1299	-3.0764	0.03704	Polymerase
1300	-4.27435	0.01054	Polymerase
1306	-4.48099	0.00867	Polymerase
1314	-3.36074	0.02841	Polymerase
1319	-3.0764	0.03704	Polymerase
1361	-2.95185	0.04359	Polymerase
1363	-3.0764	0.03704	Polymerase
1378	-3.36074	0.02841	Polymerase
1387	-3.0764	0.03704	Polymerase
1400	-6	0.00137	Polymerase
1428	-4.36046	0.0375	Polymerase
1453	-3.27728	0.02841	Polymerase
1484	-3	0.03704	Polymerase
1534	-3.80291	0.03728	Polymerase
1536	-3.27728	0.02841	Polymerase
1567	-3	0.03704	Polymerase
1569	-10.5317	0.03165	Polymerase
1572	-4	0.01235	Polymerase
1581	-3.27728	0.02841	Polymerase
1600	-3.0764	0.03704	Polymerase
1622	-10.0005	0.03418	Polymerase
1687	-4.48099	0.00867	Polymerase
1695	-4.10187	0.01235	Polymerase
1744	-3.36074	0.02841	Polymerase
1755	-3.36074	0.02841	Polymerase
1771	-3.0764	0.03799	Polymerase
1779	-3.36074	0.02841	Polymerase
1780	-3.20983	0.03265	Polymerase
1817	-3.0764	0.03704	Polymerase
1823	-3.0764	0.03704	Polymerase
1844	-4.48099	0.00867	Polymerase
1890	-3.34054	0.0301	Polymerase
1927	-3.0764	0.03704	Polymerase
1932	-4.10187	0.01235	Polymerase
1960	-11.6664	0.0293	Polymerase
1971	-3.33967	0.03012	Polymerase
1993	-5.12734	0.00412	Polymerase
1997	-3.36074	0.02841	Polymerase
2001	-3.36074	0.02841	Polymerase
2017	-3.21033	0.03269	Polymerase
2029	-3.36074	0.02841	Polymerase
2045	-3.34581	0.02995	Polymerase
28	-3.54248	0.02687	Glycoprotein
30	-3.54978	0.02675	Glycoprotein
59	-3.11896	0.03706	Glycoprotein
64	-3.34519	0.03024	Glycoprotein
115	-3.11896	0.03704	Glycoprotein
165	-3.11896	0.03736	Glycoprotein
172	-45.5896	0.0075	Glycoprotein
179	-3.54978	0.02675	Glycoprotein
198	-4.15861	0.01294	Glycoprotein
200	-3.54978	0.02512	Glycoprotein
202	-3.11896	0.03704	Glycoprotein
205	-3.11896	0.04026	Glycoprotein
211	-3.11896	0.03704	Glycoprotein
225	-5.91631	0.00216	Glycoprotein
326	-3	0.03704	Glycoprotein

346	-0.00287	0.00116	Glycoprotein
349	-3.40939	0.0269	Glycoprotein
362	-3.41125	0.02686	Glycoprotein
429	-4.5416	0.00904	Glycoprotein
447	-3.03944	0.03704	Glycoprotein
458	-4.05258	0.01242	Glycoprotein
482	-3.45928	0.02675	Glycoprotein
490	-3.03944	0.03927	Glycoprotein
509	-3.03944	0.03861	Glycoprotein
515	-4.15861	0.0135	Glycoprotein
519	-3.11896	0.03704	Glycoprotein
520	-3.11896	0.03704	Glycoprotein
522	-4.42128	0.00966	Glycoprotein
557	-4.15861	0.01235	Glycoprotein
591	-3.11896	0.03704	Glycoprotein
601	-4.15861	0.01235	Glycoprotein
624	-3.31596	0.03082	Glycoprotein
648	-3.34292	0.03055	Glycoprotein
670	-3.63879	0.04527	Glycoprotein
672	-3.31596	0.03082	Glycoprotein
705	-4.15861	0.01235	Glycoprotein
738	-3.54665	0.02685	Glycoprotein
769	-3.11896	0.03704	Glycoprotein
781	-3.11896	0.03711	Glycoprotein
793	-3.54978	0.02512	Glycoprotein
807	-3.11896	0.03704	Glycoprotein
824	-4.15861	0.01264	Glycoprotein
893	-3.54978	0.02512	Glycoprotein
923	-3.31596	0.03082	Glycoprotein
958	-3.11896	0.03704	Glycoprotein
971	-3.54978	0.02512	Glycoprotein
1001	-3.11896	0.03704	Glycoprotein
1051	-3.11896	0.03704	Glycoprotein
1090	-4.15861	0.01235	Glycoprotein
1115	-11.0793	0.01055	Glycoprotein
1116	-3.11896	0.03704	Glycoprotein
1127	-4.15861	0.01242	Glycoprotein
1128	-3.11896	0.03721	Glycoprotein
1141	-3.54978	0.02512	Glycoprotein
1159	-3.11896	0.03704	Glycoprotein
1184	-3.11896	0.03704	Glycoprotein
1191	-3.11896	0.03704	Glycoprotein
1192	-3.11896	0.03704	Glycoprotein
56	-3.37716	0.02596	Nucleocapsid
88	-0.00013	0.00182	Nucleocapsid
116	-5	0.00464	Nucleocapsid
119	-3	0.03992	Nucleocapsid
127	-3	0.03712	Nucleocapsid
142	-4	0.01235	Nucleocapsid
148	-3.37716	0.02596	Nucleocapsid
151	-3.75137	0.03667	Nucleocapsid
170	-3	0.03704	Nucleocapsid
192	-3.49253	0.04557	Nucleocapsid
254	-4.78769	0.00751	Nucleocapsid
76	-3	0.03928	Non-structural
90	-3.34013	0.02977	Non-structural
95	-3	0.03712	Non-structural

97	-3	0.03704	Non-structural
186	-3	0.03704	Non-structural
209	-9.60123	0.03472	Non-structural
226	-4	0.01235	Non-structural
232	-3	0.03704	Non-structural
240	-3	0.03704	Non-structural

Table S2. Akaike's information estimated by Markov chain Monte Carlo (AICM) and path-sampling tests for different prior propositions to explain evolution of RVFV strains.

Segment	AICM	S.E.	Diference between AICM	marginal log(L) from PS	Mean rate (m)	HPD
Long	41130.913	0.455	4.107	-13207.78	2.31E-04	(1.88E-4 to 2.72E-4)
Long	41135.021	0.259	-4.107	-18826.17	3.28E-04	(2.56E-4 to 3.98E-4)
Medium	27870.334	0.298	6.654	-14206.09	3.80E-04	(2.53E-4 to 5.05E-4)
Medium	27876.988	0.199	-6.654	-14208.76	2.00E-04	(1.52E-4 to 2.4E-4)
Small	11867.499	0.31	17.718	-5687.28	3.36E-04	(2.42E-4 to 4.26E-4)
Small	11885.217	0.217	-17.718	-5413.06	2.10E-04	(1.66E-4 to 2.47e-4)

Table S3. RVFV strains used in this study.

Strain name	Locality of isolation	Collection date	Accession number	Host	Segment
ARD38388	BurkinaFaso	1983	DQ375399.1	mosquito	Long
73HB1230	CentralAfricanRepublic	1973	DQ375425.1	human	Long
73HB1449	CentralAfricanRepublic	1974	DQ375416.1	human	Long
74HB59	CentralAfricanRepublic	1974	DQ375415.1	human	Long
Zinga	CentralAfricanRepublic	1969	DQ375419.1	human	Long
CARR1622	CentralAfricanRepublic$Bangui	1985	DQ375423.1	human	Long
HvB375	CentralAfricanRepublic$Mbaki	1985	DQ375422.1	human	Long
ZC3349	Egypt$Asyut	1978	DQ375412.1	bovine	Long
ZH1776	Egypt$Gharbiya	1978	DQ375411.1	human	Long
ZS6365	Egypt$Gharbiya	1979	DQ375410.1	ovine	Long
T46228113	Egypt$Sharqiya	1977	DQ375405.1	mosquito	Long
T1	Egypt$Sharqiya	1977	DQ375407.1	mosquito	Long
ZH501	Egypt$Sharqiya	1977	DQ375406.1	human	Long
ZH548	Egypt$Sharqiya	1977	DQ375403.1	human	Long
ZM657	Egypt$Sharqiya	1978	DQ375409.1	mosquito	Long
ZH501777	Egypt$Sharqiya	1977	DQ375408.1	plaque pick of RVFV ZH-501	Long
ANK3837	Guinea	1981	DQ375420.1	bat	Long
ANK6087	Guinea	1984	DQ375421.1	bat	Long
2007000234	Kenya	2007	JF326186.1	human	Long
KEN/Bar032/07	Kenya	3-Feb-07	HM586957.1	human	Long
KEN/Bar035/07	Kenya	30-Jan-07	HM586958.1	human	Long
KEN/Gar004/06	Kenya	21-Dec-06	HM586953.1	human	Long
KEN/Gar008/06	Kenya	21-Dec-06	HM586954.1	human	Long
KEN/Kil006/07	Kenya	5-Jan-07	HM586955.1	human	Long
KEN/Mal032/07	Kenya	15-Jan-07	HM586956.1	human	Long
Kenya9800523	Kenya	1998	DQ375400.1	human	Long
Kenya57Rintoul	Kenya	1951	DQ375431.1	ovine	Long
2007003644	Kenya$Baringodistrict	7-Feb-07	EU574006.1	ovine	Long
2007001809	Kenya$Garissadisrict	1-Dec-06	EU574015.1	caprine	Long
2007000094	Kenya$Garissadistrict	12-Jan-07	EU574029.1	bovine	Long
2007001811	Kenya$Garissadistrict	1-Dec-06	EU574014.1	caprine	Long
2007000473	Kenya$Kajaidodistrict	26-Jan-07	EU574024.1	ovine	Long
2007004194	Kenya$Kiambudistrict	14-May-07	EU574004.1	bovine	Long
2007000080	Kenya$Maraguadistrict	12-Jan-07	EU574030.1	bovine	Long
2007000222	Kenya$Maraguadistrict	16-Jan-07	EU574028.1	bovine	Long
2007000223	Kenya$Maraguadistrict	16-Jan-07	EU574027.1	bovine	Long
2007000224	Kenya$Maraguadistrict	16-Jan-07	EU574026.1	bovine	Long
2007000225	Kenya$Maraguadistrict	16-Jan-07	EU574025.1	bovine	Long
2007003081	Kenya$Mbeeredistrict	27-Feb-07	EU574007.1	bovine	Long
2007002444	Kenya$MeruCentraldistrict	5-Feb-07	EU574012.1	bovine	Long
2007002445	Kenya$MeruCentraldistrict	5-Feb-07	EU574011.1	bovine	Long
2007000608	Kenya$MeruSouthdistrict	29-Jan-07	EU574023.1	bovine	Long
2007000611	Kenya$MeruSouthdistrict	29-Jan-07	EU574022.1	bovine	Long
2007000618	Kenya$MeruSouthdistrict	29-Jan-07	EU574021.1	bovine	Long
2007001292	Kenya$MeruSouthdistrict	31-Jan-07	EU574019.1	bovine	Long
2007001602	Kenya$Mombassadistrict	30-Jan-07	EU574016.1	bovine	Long
2007001564	Kenya$Murangadistrict	29-Jan-07	EU574017.1	bovine	Long
2007002060	Kenya$Nairobidistrict	31-Jan-07	EU574013.1	bovine	Long
2007004193	Kenya$Nairobidistrict	23-Apr-07	EU574005.1	bovine	Long
2007001443	Kenya$Nakurudistrict	31-Jan-07	EU574018.1	bovine	Long
2007002476	Kenya$Nakurudistrict	21-Feb-07	EU574010.1	bovine	Long
Kenya8321445	Kenya$Ruiru	1983	DQ375402.1	mosquito	Long
2007001107	Kenya$Thikadistrict	25-Jan-07	EU574020.1	bovine	Long
2007002482	Kenya$Thikadistrict	21-Feb-07	EU574009.1	bovine	Long
40608	Madagascar	20-Feb-08	GQ443177.1	bovine	Long
41308	Madagascar	20-Feb-08	GQ443178.1	bovine	Long
85508	Madagascar	24-Mar-08	GQ443200.1	bovine	Long
162708	Madagascar	18-Apr-08	GQ443212.1	bovine	Long
173008	Madagascar	25-Apr-08	GQ443214.1	bovine	Long
158508	Madagascar	13-Apr-08	GQ443210.1	caprine	Long
21208	Madagascar	30-Jan-08	GQ443176.1	human	Long
42708	Madagascar	22-Feb-08	GQ443179.1	human	Long
42808	Madagascar	15-Feb-08	GQ443180.1	human	Long
44808	Madagascar	26-Feb-08	GQ443182.1	human	Long
61908	Madagascar	9-Mar-08	GQ443184.1	human	Long
68208	Madagascar	12-Mar-08	GQ443185.1	human	Long
68308	Madagascar	12-Mar-08	GQ443186.1	human	Long
68408	Madagascar	12-Mar-08	GQ443187.1	human	Long
69308	Madagascar	11-Mar-08	GQ443188.1	human	Long
77608	Madagascar	14-Mar-08	GQ443189.1	human	Long
84508	Madagascar	23-Mar-08	GQ443191.1	human	Long
84608	Madagascar	23-Mar-08	GQ443192.1	human	Long
84708	Madagascar	23-Mar-08	GQ443193.1	human	Long
84808	Madagascar	23-Mar-08	GQ443194.1	human	Long
84908	Madagascar	23-Mar-08	GQ443195.1	human	Long
85108	Madagascar	23-Mar-08	GQ443196.1	human	Long
85208	Madagascar	23-Mar-08	GQ443197.1	human	Long
85308	Madagascar	23-Mar-08	GQ443198.1	human	Long
85408	Madagascar	23-Mar-08	GQ443199.1	human	Long
86308	Madagascar	25-Mar-08	GQ443203.1	human	Long

87808	Madagascar	26-Mar-08	GQ443204.1	human	Long
87908	Madagascar	26-Mar-08	GQ443205.1	human	Long
89208	Madagascar	26-Mar-08	GQ443206.1	human	Long
89508	Madagascar	26-Mar-08	GQ443207.1	human	Long
89708	Madagascar	26-Mar-08	GQ443208.1	human	Long
146408	Madagascar	28-Mar-08	GQ443209.1	human	Long
158608	Madagascar	15-Apr-08	GQ443211.1	human	Long
203208	Madagascar	25-May-08	GQ443215.1	human	Long
203308	Madagascar	29-Apr-08	GQ443216.1	human	Long
203408	Madagascar	23-Apr-08	GQ443217.1	human	Long
MgH824	Madagascar	1979	DQ375414.1	human	Long
169508	Madagascar	20-Apr-08	GQ443213.1	ovine	Long
200803163	Madagascar$Anjozorobe	Feb-08	JF311369.1	human	Long
200803164	Madagascar$Ankazobe	Mar-08	JF311370.1	human	Long
200803167	Madagascar$Antananarivo	Feb-91	JF311373.1	bovine	Long
200803166	Madagascar$Antananarivo	Feb-91	JF311372.1	human	Long
200803169	Madagascar$Antsirabe	Feb-08	JF311375.1	bovine	Long
200803170	Madagascar$Manjakandriana	Mar-08	JF311376.1	bovine	Long
200803165	Madagascar$Manjakandriana	Mar-08	JF311371.1	human	Long
200803168	Madagascar$Miarinarivo	Feb-08	JF311374.1	bovine	Long
200803162	Madagascar$Taolagnaro	Jan-08	JF311368.1	human	Long
OS1	Mauritania	1987	DQ375398.1	human	Long
OS3	Mauritania	1987	DQ375396.1	human	Long
OS8	Mauritania	1987	DQ375395.1	human	Long
OS9	Mauritania	1987	DQ375397.1	human	Long
2008/00099	Mayotte	21-Feb-08	HE687304.1	human	Long
2008/00101	Mayotte	20-Mar-08	HE687305.1	human	Long
Saudi200010911	SaudiArabia	2000	DQ375401.1	human	Long
35/74	SouthAfrica	1974	JF784386.1	sheep	Long
Kakamas	SouthAfrica	Oct-09	JQ068144.1	sheep	Long
SA51VanWyck	SouthAfrica$Boshof	1951	DQ375433.1	ovine	Long
SA75	SouthAfrica$Randfontein	1975	DQ375428.1	human	Long
Sudan282010	Sudan$GeziraState	Oct-10	JQ820486.1	human	Long
Sudan852010	Sudan$GeziraState	Oct-10	JQ820485.1	human	Long
Sudan862010	Sudan$GeziraState	Oct-10	JQ820484.1	human	Long
Sudan2V2007	Sudan$WhiteNileState	2007	JQ820483.1	human	Long
2007000323	Tanzania	Feb-07	JF326189.1	human	Long
2007000324	Tanzania	Feb-07	JF326190.1	human	Long
TAN/Dod002/07	Tanzania	14-Mar-07	HM586960.1	human	Long
TAN/Tan001/07	Tanzania	8-Feb-07	HM586959.1	human	Long
Entebbe	Uganda	1944	DQ375429.1	mosquito	Long
2250/74	Zimbabwe$Beatrice	1974	DQ375413.1	bovine	Long
1260/78	Zimbabwe$Salisbury	1978	DQ375418.1	bovine	Long
2373/74	Zimbabwe$Salisbury	1974	DQ375432.1	bovine	Long
763/70	Zimbabwe$Salisbury	1970	DQ375426.1	bovine	Long
1853/78	Zimbabwe$Sinoia	1978	DQ375424.1	bovine	Long
2269/74	Zimbabwe$Sinoia	1974	DQ375434.1	bovine	Long
ARD38388	BurkinaFaso	1983	DQ380187.1	mosquito	Medium
73HB1230	CentralAfricanRepublic	1973	DQ380221.1	human	Medium
73HB1449	CentralAfricanRepublic	1973	DQ380211.1	human	Medium
74HB59	CentralAfricanRepublic	1974	HM587082.1	human	Medium
Zinga	CentralAfricanRepublic	1969	DQ380217.1	mosquito	Medium
HvB375	CentralAfricanRepublic$Abaki	7-Jun-05	DQ380218.1	human	Medium
CARR1622	CentralAfricanRepublic$Bangui	7-Jun-05	DQ380219.1	human	Medium
T1	Egypt	1977	DQ380201.1	mosquito	Medium
T46228113	Egypt	1977	DQ380199.1	mosquito	Medium
ZH501777	Egypt	1977	DQ380202.1	plaque pick of ZH-501	Medium
ZC3349	Egypt$Asyut	31-May-05	DQ380207.1	bovine	Medium
ZH1776	Egypt$Gharbiya	31-May-05	DQ380203.1	human	Medium
ZS6365	Egypt$Gharbiya	1-Jun-05	DQ380205.1	ovine	Medium
ZH501	Egypt$Sharqiya	30-May-05	DQ380200.1	human	Medium
ZH548	Egypt$Sharqiya	30-May-05	DQ380206.1	human	Medium
ZM657	Egypt$Sharqiya	31-May-05	DQ380204.1	mosquito	Medium
ANK3837	Guinea	3-Jun-05	DQ380215.1	bat	Medium
ANK6087	Guinea	6-Jun-05	DQ380216.1	bat	Medium
2007000234	Kenya	29-Jun-05	JF326191.1	human	Medium
2007000253	Kenya	29-Dec-06	JF326192.1	mosquito	Medium
KEN/Bar032/07	Kenya	3-Feb-07	HM586968.1	Unknown	Medium
KEN/Bar035/07	Kenya	30-Jan-07	HM586969.1	Unknown	Medium
KEN/Gar004/06	Kenya	21-Dec-06	HM586964.1	Unknown	Medium
KEN/Gar008/06	Kenya	21-Dec-06	HM586965.1	Unknown	Medium
KEN/Kil006/07	Kenya	5-Jan-07	HM586966.1	Unknown	Medium
KEN/Mal032/07	Kenya	15-Jan-07	HM586967.1	Unknown	Medium
Kenya9800523	Kenya	1998	DQ380196.1	human	Medium
2007003644	Kenya$Baringodistrict	7-Feb-07	EU574033.1	ovine	Medium
2007001809	Kenya$Garissadisrict	1-Dec-06	EU574041.1	caprine	Medium
2007000094	Kenya$Garissadistrict	12-Jan-07	EU574055.1	bovine	Medium
2007001811	Kenya$Garissadistrict	1-Dec-06	EU574040.1	caprine	Medium
2007000473	Kenya$Kajaidodistrict	26-Jan-07	EU574050.1	ovine	Medium
2007004194	Kenya$Kiambudistrict	14-May-07	EU574031.1	bovine	Medium
Kenya57Rintoul	Kenya$Kitale	1951	DQ380192.1	ovine	Medium

2007000080	Kenya$Maraguadistrict	12-Jan-07	EU574056.1	bovine	Medium
2007000222	Kenya$Maraguadistrict	16-Jan-07	EU574054.1	bovine	Medium
2007000223	Kenya$Maraguadistrict	16-Jan-07	EU574053.1	bovine	Medium
2007000224	Kenya$Maraguadistrict	16-Jan-07	EU574052.1	bovine	Medium
2007000225	Kenya$Maraguadistrict	16-Jan-07	EU574051.1	bovine	Medium
2007003081	Kenya$Mbeeredistrict	27-Feb-07	EU574034.1	bovine	Medium
2007002444	Kenya$MeruCentraldistrict	5-Feb-07	EU574038.1	bovine	Medium
2007002445	Kenya$MeruCentraldistrict	5-Feb-07	EU574037.1	bovine	Medium
2007000608	Kenya$MeruSouthdistrict	29-Jan-07	EU574049.1	bovine	Medium
2007000611	Kenya$MeruSouthdistrict	29-Jan-07	EU574048.1	bovine	Medium
2007000618	Kenya$MeruSouthdistrict	29-Jan-07	EU574042.1	bovine	Medium
2007001292	Kenya$MeruSouthdistrict	31-Jan-07	EU574046.1	bovine	Medium
2007001602	Kenya$Mombassadistrict	30-Jan-07	EU574043.1	bovine	Medium
2007001564	Kenya$Murangadistrict	29-Jan-07	EU574044.1	bovine	Medium
2007002060	Kenya$Nairobidistrict	31-Jan-07	EU574039.1	bovine	Medium
2007004193	Kenya$Nairobidistrict	23-Apr-07	EU574032.1	bovine	Medium
2007001443	Kenya$Nakurudistrict	31-Jan-07	EU574045.1	bovine	Medium
2007002476	Kenya$Nakurudistrict	21-Feb-07	EU574036.1	bovine	Medium
Kenya8321445	Kenya$Ruiru	1983	DQ380198.1	mosquito	Medium
2007001107	Kenya$Thikadistrict	25-Jan-07	EU574047.1	bovine	Medium
2007002482	Kenya$Thikadistrict	21-Feb-07	EU574035.1	bovine	Medium
21208	Madagascar	30-Jan-08	GQ443218.1	human	Medium
40608	Madagascar	Feb-08	GQ443219.1	bovine	Medium
41308	Madagascar	Feb-08	GQ443220.1	bovine	Medium
42708	Madagascar	22-Feb-08	GQ443221.1	human	Medium
42808	Madagascar	15-Feb-08	GQ443222.1	human	Medium
44808	Madagascar	26-Feb-08	GQ443223.1	human	Medium
61908	Madagascar	Mar-08	GQ443224.1	human	Medium
68208	Madagascar	12-Mar-08	GQ443225.1	human	Medium
68308	Madagascar	12-Mar-08	GQ443226.1	human	Medium
68408	Madagascar	12-Mar-08	GQ443227.1	human	Medium
69308	Madagascar	Mar-08	GQ443228.1	human	Medium
77608	Madagascar	14-Mar-08	GQ443229.1	human	Medium
84508	Madagascar	23-Mar-08	GQ443230.1	human	Medium
84608	Madagascar	23-Mar-08	GQ443231.1	human	Medium
84708	Madagascar	23-Mar-08	GQ443232.1	human	Medium
84808	Madagascar	23-Mar-08	GQ443233.1	human	Medium
84908	Madagascar	23-Mar-08	GQ443234.1	human	Medium
85008	Madagascar	Mar-08	GQ443235.1	human	Medium
85108	Madagascar	23-Mar-08	GQ443236.1	human	Medium
85208	Madagascar	23-Mar-08	GQ443237.1	human	Medium
85308	Madagascar	23-Mar-08	GQ443238.1	human	Medium
85408	Madagascar	23-Mar-08	GQ443239.1	human	Medium
85508	Madagascar	24-Mar-08	GQ443240.1	bovine	Medium
86308	Madagascar	25-Mar-08	GQ443241.1	human	Medium
87808	Madagascar	26-Mar-08	GQ443242.1	human	Medium
87908	Madagascar	26-Mar-08	GQ443243.1	human	Medium
88908	Madagascar	26-Mar-08	GQ443244.1	human	Medium
89208	Madagascar	26-Mar-08	GQ443245.1	human	Medium
89508	Madagascar	26-Mar-08	GQ443246.1	human	Medium
89708	Madagascar	26-Mar-08	GQ443247.1	human	Medium
146408	Madagascar	Mar-08	GQ443248.1	human	Medium
158508	Madagascar	Apr-08	GQ443249.1	caprine	Medium
158608	Madagascar	15-Apr-08	GQ443250.1	human	Medium
162708	Madagascar	18-Apr-08	GQ443251.1	bovine	Medium
169508	Madagascar	20-Apr-08	GQ443252.1	ovine	Medium
173008	Madagascar	Apr-08	GQ443253.1	bovine	Medium
203208	Madagascar	May-08	GQ443254.1	human	Medium
203308	Madagascar	29-Apr-08	GQ443255.1	human	Medium
203408	Madagascar	23-Apr-08	GQ443256.1	human	Medium
MgH824	Madagascar	1-Jun-05	HM587040.1	human	Medium
FI28	Madagascar$AmpandrambatoSahambavy	19-Dec-08	GU135857.1	mosquito	Medium
200803163	Madagascar$Anjozorobe	Feb-08	JF311378.1	human	Medium
200803164	Madagascar$Ankazobe	1-Mar-08	JF311379.1	human	Medium
200803166	Madagascar$Antananarivo	Feb-91	JF311381.1	human	Medium
200803167	Madagascar$Antananarivo	Feb-91	JF311382.1	bovine	Medium
FI148	Madagascar$AntanifotsyFianarantsoaI	19-Dec-08	GU135859.1	mosquito	Medium
200803169	Madagascar$Antsirabe	1-Feb-08	JF311384.1	bovine	Medium
200803165	Madagascar$Manjakandriana	Mar-08	JF311380.1	human	Medium
200803170	Madagascar$Manjakandriana	Mar-08	JF311385.1	bovine	Medium
200803168	Madagascar$Miarinarivo	1-Feb-08	JF311383.1	bovine	Medium
200803162	Madagascar$Taolagnaro	1-Jan-08	JF311377.1	human	Medium
211977	Mauritania	5-Dec-10	KF717590.1	human	Medium
227595	Mauritania	2-Nov-12	KF648851.1	human	Medium
227598	Mauritania	2-Nov-12	KF648852.1	human	Medium
227601	Mauritania	2-Nov-12	KF648853.1	human	Medium
227602	Mauritania	2-Nov-12	KF648854.1	human	Medium
227603	Mauritania	2-Nov-12	KF648855.1	human	Medium
227608	Mauritania	2-Nov-12	KF648856.1	human	Medium
OS1	Mauritania	1987	DQ380186.1	human	Medium
OS3	Mauritania	1987	DQ380184.1	human	Medium

OS8	Mauritania	1987	DQ380185.1	human	Medium
OS9	Mauritania	1987	DQ380183.1	human	Medium
M30ARMcxMRAA1999	Mauritania$AyounElAtross	1999	JN995322.1	mosquito	Medium
M214HMMRGA1987	Mauritania$Garack	1987	JN995303.1	human	Medium
M298ARMcxMRGI2003	Mauritania$Guimi	2003	JN995336.1	mosquito	Medium
M303ARMcxMRGI2003	Mauritania$Guimi	2003	JN995337.1	mosquito	Medium
M347ARMcxMRGI2003	Mauritania$Guimi	2003	JN995338.1	mosquito	Medium
M356ARMcxMRGI2003	Mauritania$Guimi	2003	JN995339.1	mosquito	Medium
M11ANMMRHG1998	Mauritania$HodhElGarbi	1998	JN995312.1	sheep	Medium
M16ANMMRHG1998	Mauritania$HodhElGarbi	1998	JN995313.1	Unknown	Medium
M22ANMMRHG1998	Mauritania$HodhElGarbi	1998	JN995314.1	Unknown	Medium
M24ANMMRHG1998	Mauritania$HodhElGarbi	1998	JN995315.1	sheep	Medium
M25ANMMRHG1998	Mauritania$HodhElGarbi	1998	JN995316.1	sheep	Medium
M292HMMRHG1998	Mauritania$HodhElGarbi	1998	JN995344.1	human	Medium
M208HMMRKM1987	Mauritania$KeurMacene	1987	JN995300.1	human	Medium
M226HMMRNK1987	Mauritania$Nkick	1987	JN995302.1	human	Medium
M211HMMRRO1987	Mauritania$Rosso	1987	JN995301.1	human	Medium
M232HMMRRO1987	Mauritania$Rosso	1987	JN995305.1	human	Medium
M209HMMRTG1987	Mauritania$Tegmaline	1987	JN995345.1	human	Medium
M223HMMRTE1987	Mauritania$Terg	1987	JN995304.1	human	Medium
2008/00099	Mayotte	21-Feb-08	HE687303.1	human	Medium
2008/00101	Mayotte	20-Mar-08	HE687306.1	human	Medium
Saudi200010911	SaudiArabia	2000	DQ380197.1	human	Medium
M18ANBkSNBA1993	Senegal$Barkedji	1993	JN995309.1	sheep	Medium
M31ARBkcxSNBA2002	Senegal$Barkedji	2002	JN995324.1	mosquito	Medium
M32ARBkcxSNBA2002	Senegal$Barkedji	Jun-05	JN995325.1	mosquito	Medium
M33ARBkcxSNBA2002	Senegal$Barkedji	Jun-05	JN995326.1	mosquito	Medium
M35ARBkcxSNBA2003	Senegal$Barkedji	2003	JN995340.1	mosquito	Medium
M36ARBkcxSNBA2003	Senegal$Barkedji	2003	JN995341.1	mosquito	Medium
M10ARDwcxSNDI1998	Senegal$Diawara	1998	JN995320.1	mosquito	Medium
M27ARDwcxSNDI1998	Senegal$Diawara	1998	JN995317.1	mosquito	Medium
M28ARDwcxSNDI1998	Senegal$Diawara	1998	JN995318.1	mosquito	Medium
M9ARDwcxSNDI1998	Senegal$Diawara	1998	JN995319.1	mosquito	Medium
M233HMMRRO1987	Senegal$Rosso	1987	JN995306.1	human	Medium
35/74	SouthAfrica	1974	JF784387.1	sheep	Medium
Kakamas	SouthAfrica	Oct-09	JQ068143.1	sheep	Medium
SA51VanWyck	SouthAfrica$Boshof	1951	DQ380195.1	ovine	Medium
SA75	SouthAfrica$Randfontein	1975	DQ380189.1	human	Medium
Sudan282010	Sudan$GeziraState	1-Oct-10	JQ820491.1	human	Medium
Sudan72010	Sudan$GeziraState	1-Oct-10	JQ820487.1	human	Medium
Sudan852010	Sudan$GeziraState	Oct-10	JQ820488.1	human	Medium
Sudan862010	Sudan$GeziraState	Oct-10	JQ820489.1	human	Medium
Sudan2V2007	Sudan$WhiteNileState	29-Jun-05	JQ820490.1	human	Medium
2007000323	Tanzania	1-Feb-07	JF326194.1	human	Medium
2007000324	Tanzania	Feb-07	JF326195.1	human	Medium
TAN/Dod002/07	Tanzania	14-Mar-07	HM586971.1	Unknown	Medium
TAN/Tan001/07	Tanzania	8-Feb-07	HM586970.1	Unknown	Medium
Entebbe	Uganda	1944	DQ380191.1	mosquito	Medium
2250/74	Zimbabwe$Beatrice	27-May-05	DQ380209.1	bovine	Medium
1260/78	Zimbabwe$Salisbury	1978	DQ380214.1	bovine	Medium
2373/74	Zimbabwe$Salisbury	1974	DQ380194.1	bovine	Medium
763/70	Zimbabwe$Salisbury	1970	DQ380188.1	bovine	Medium
1853/78	Zimbabwe$Sinoia	31-May-05	DQ380220.1	bovine	Medium
2269/74	Zimbabwe$Sinoia	27-May-05	DQ380222.1	bovine	Medium
ANK3837	Guinea	3-Jun-05	DQ380165.1	bat	Small
ANK6087	Guinea	6-Jun-05	DQ380166.1	bat	Small
40608	Madagascar	Feb-08	GQ443127.1	bovine	Small
41308	Madagascar	Feb-08	GQ443128.1	bovine	Small
43508	Madagascar	22-Feb-08	GQ443131.1	bovine	Small
85508	Madagascar	24-Mar-08	GQ443152.1	bovine	Small
85608	Madagascar	24-Mar-08	GQ443153.1	bovine	Small
85908	Madagascar	24-Mar-08	GQ443155.1	bovine	Small
162708	Madagascar	18-Apr-08	GQ443166.1	bovine	Small
173008	Madagascar	Apr-08	GQ443168.1	bovine	Small
200803167	Madagascar$Antananarivo	Feb-91	JF311391.1	bovine	Small
200803168	Madagascar$Miarinarivo	1-Feb-08	JF311392.1	bovine	Small
200803169	Madagascar$Antsirabe	1-Feb-08	JF311393.1	bovine	Small
2007000080	Kenya$Maraguadistrict	12-Jan-07	EU574087.1	bovine	Small
2007000094	Kenya$Garissadistrict	12-Jan-07	EU574086.1	bovine	Small
2007000222	Kenya$Maraguadistrict	16-Jan-07	EU574085.1	bovine	Small
2007000223	Kenya$Maraguadistrict	16-Jan-07	EU574084.1	bovine	Small
2007000224	Kenya$Maraguadistrict	16-Jan-07	EU574083.1	bovine	Small
2007000225	Kenya$Maraguadistrict	16-Jan-07	EU574082.1	bovine	Small
2007000608	Kenya$MeruSouthdistrict	29-Jan-07	EU574079.1	bovine	Small
2007000611	Kenya$MeruSouthdistrict	29-Jan-07	EU574078.1	bovine	Small
2007000618	Kenya$MeruSouthdistrict	29-Jan-07	EU574077.1	bovine	Small
2007001107	Kenya$Thikadistrict	25-Jan-07	EU574075.1	bovine	Small
2007001292	Kenya$MeruSouthdistrict	31-Jan-07	EU574074.1	bovine	Small
2007001443	Kenya$Nakurudistrict	31-Jan-07	EU574073.1	bovine	Small
2007001564	Kenya$Murangadistrict	29-Jan-07	EU574072.1	bovine	Small
2007001602	Kenya$Mombassadistrict	30-Jan-07	EU574071.1	bovine	Small

2007002060	Kenya$Nairobidistrict	31-Jan-07	EU574066.1	bovine	Small
2007002444	Kenya$MeruCentraldistrict	5-Feb-07	EU574065.1	bovine	Small
2007002445	Kenya$MeruCentraldistrict	5-Feb-07	EU574064.1	bovine	Small
2007002476	Kenya$Nakurudistrict	21-Feb-07	EU574063.1	bovine	Small
2007002482	Kenya$Thikadistrict	21-Feb-07	EU574062.1	bovine	Small
2007003081	Kenya$Mbeeredistrict	27-Feb-07	EU574060.1	bovine	Small
2007004193	Kenya$Nairobidistrict	23-Apr-07	EU574058.1	bovine	Small
2007004194	Kenya$Kiambudistrict	14-May-07	EU574057.1	bovine	Small
1260/78	Zimbabwe$Salisbury	1978	DQ380164.1	bovine	Small
1853/78	Zimbabwe$Sinoia	31-May-05	DQ380168.1	bovine	Small
2250/74	Zimbabwe$Beatrice	27-May-05	DQ380143.1	bovine	Small
2269/74	Zimbabwe$Sinoia	27-May-05	DQ380173.1	bovine	Small
2373/74	Zimbabwe$Salisbury	1974	DQ380159.1	bovine	Small
763/70	Zimbabwe$Salisbury	1970	DQ380174.1	bovine	Small
ZC3349	Egypt$Asyut	31-May-05	DQ380152.1	bovine	Small
158508	Madagascar	Apr-08	GQ443164.1	caprine	Small
2007001809	Kenya$Garissadisrict	1-Dec-06	EU574069.1	caprine	Small
2007001811	Kenya$Garissadistrict	1-Dec-06	EU574068.1	caprine	Small
21208	Madagascar	30-Jan-08	GQ443126.1	human	Small
42708	Madagascar	Feb-08	GQ443129.1	human	Small
42808	Madagascar	15-Feb-08	GQ443130.1	human	Small
44808	Madagascar	26-Feb-08	GQ443133.1	human	Small
44908	Madagascar	26-Feb-08	GQ443134.1	human	Small
61908	Madagascar	Mar-08	GQ443135.1	human	Small
68208	Madagascar	12-Mar-08	GQ443136.1	human	Small
68308	Madagascar	12-Mar-08	GQ443137.1	human	Small
68408	Madagascar	12-Mar-08	GQ443138.1	human	Small
69308	Madagascar	Mar-08	GQ443139.1	human	Small
77608	Madagascar	14-Mar-08	GQ443140.1	human	Small
77908	Madagascar	16-Mar-08	GQ443141.1	human	Small
84508	Madagascar	23-Mar-08	GQ443142.1	human	Small
84608	Madagascar	23-Mar-08	GQ443143.1	human	Small
84708	Madagascar	23-Mar-08	GQ443144.1	human	Small
84808	Madagascar	23-Mar-08	GQ443145.1	human	Small
84908	Madagascar	23-Mar-08	GQ443146.1	human	Small
85008	Madagascar	Mar-08	GQ443147.1	human	Small
85108	Madagascar	23-Mar-08	GQ443148.1	human	Small
85208	Madagascar	23-Mar-08	GQ443149.1	human	Small
85308	Madagascar	23-Mar-08	GQ443150.1	human	Small
85408	Madagascar	23-Mar-08	GQ443151.1	human	Small
86308	Madagascar	25-Mar-08	GQ443156.1	human	Small
87808	Madagascar	26-Mar-08	GQ443157.1	human	Small
87908	Madagascar	26-Mar-08	GQ443158.1	human	Small
88908	Madagascar	26-Mar-08	GQ443159.1	human	Small
89208	Madagascar	26-Mar-08	GQ443160.1	human	Small
89508	Madagascar	26-Mar-08	GQ443161.1	human	Small
89708	Madagascar	26-Mar-08	GQ443162.1	human	Small
146408	Madagascar	Mar-08	GQ443163.1	human	Small
158608	Madagascar	Apr-08	GQ443165.1	human	Small
203208	Madagascar	25-Apr-08	GQ443169.1	human	Small
203308	Madagascar	29-Apr-08	GQ443170.1	human	Small
203408	Madagascar	23-Apr-08	GQ443171.1	human	Small
211977	Mauritania	5-Dec-10	KF717593.1	human	Small
227595	Mauritania	2-Nov-12	KF648857.1	human	Small
227598	Mauritania	2-Nov-12	KF648858.1	human	Small
227601	Mauritania	2-Nov-12	KF648859.1	human	Small
227602	Mauritania	2-Nov-12	KF648860.1	human	Small
227603	Mauritania	2-Nov-12	KF648861.1	human	Small
227608	Mauritania	2-Nov-12	KF648862.1	human	Small
200803162	Madagascar$Taolagnaro	1-Jan-08	JF311386.1	human	Small
200803164	Madagascar$Ankazobe	1-Mar-08	JF311388.1	human	Small
200803166	Madagascar$Antananarivo	Feb-91	JF311390.1	human	Small
2007000234	Kenya	29-Jun-05	JF326198.1	human	Small
2007000323	Tanzania	1-Feb-07	JF326203.1	human	Small
2008/00099	Mayotte	21-Feb-08	HE687302.1	human	Small
2008/00101	Mayotte	20-Mar-08	HE687307.1	human	Small
73HB1230	CentralAfricanRepublic	1973	DQ380172.1	human	Small
73HB1449	CentralAfricanRepublic	1973	DQ380162.1	human	Small
74HB59	CentralAfricanRepublic	1974	DQ380163.1	human	Small
CARR1622	CentralAfricanRepublic	7-Jun-05	DQ380160.1	human	Small
HvB375	CentralAfricanRepublic	7-Jun-05	DQ380161.1	human	Small
Kenya9800523	Kenya	1998	DQ380169.1	human	Small
MgH824	Madagascar	1-Jun-05	DQ380144.1	human	Small
OS1	Mauritania	1987	DQ380180.1	human	Small
OS3	Mauritania	1987	DQ380178.1	human	Small
OS8	Mauritania	1987	DQ380177.1	human	Small
OS9	Mauritania	1987	DQ380179.1	human	Small
S208HMMRKM1987	Mauritania$KeurMacene	1987	JN995252.1	human	Small
S209HMMRTG1987	Mauritania$Tegmaline	1987	JN995298.1	human	Small
S211HMMRRO1987	Mauritania$Rosso	1987	JN995253.1	human	Small
S214HMMRGA1987	Mauritania$Garack	1987	JN995255.1	human	Small

S223HMMRTE1987	Mauritania$Terg	1987	JN995256.1	human	Small
S226HMMRNK1987	Mauritania$Nkick	1987	JN995254.1	human	Small
S232HMMRRO1987	Mauritania$Rosso	1987	JN995257.1	human	Small
S233HMMRRO1987	Mauritania$Rosso	1987	JN995258.1	human	Small
S292HMMRHG1998	Mauritania$HodhElGarbi	1998	JN995297.1	human	Small
SA75	SouthAfrica$Randfontein	1975	DQ380175.1	human	Small
Saudi200010911	SaudiArabia	2000	DQ380170.1	human	Small
Sudan282010	Sudan$GeziraState	1-Oct-10	JQ820474.1	human	Small
Sudan2V2007	Sudan$WhiteNileState	29-Jun-05	JQ820472.1	human	Small
Sudan72010	Sudan$GeziraState	1-Oct-10	JQ820480.1	human	Small
Sudan852010	Sudan$GeziraState	1-Oct-10	JQ820476.1	human	Small
Sudan862010	Sudan$GeziraState	Oct-10	JQ820477.1	human	Small
ZH1776	Egypt$Gharbiya	31-May-05	DQ380153.1	human	Small
ZH501	Egypt	30-May-05	GU372973.1	human	Small
ZH548	Egypt$Sharqiya	30-May-05	DQ380151.1	human	Small
2007000253	Kenya	29-Dec-06	JF326201.1	mosquito	Small
ARD38388	BurkinaFaso	1983	DQ380181.1	mosquito	Small
Entebbe	Uganda	1944	DQ380156.1	mosquito	Small
FI148	Madagascar$AntanifotsyFianarantsoaI	Dec-08	GU135843.1	mosquito	Small
FI28	Madagascar$AmpandrambatoSahambavy	Dec-08	GU135841.1	mosquito	Small
Kenya8321445	Kenya	1983	DQ380171.1	mosquito	Small
S10ARDwcxSNDI1998	Senegal$Diawara	1998	JN995272.1	mosquito	Small
S27ARDwcxSNDI1998	Senegal$Diawara	1998	JN995269.1	mosquito	Small
S28ARDwcxSNDI1998	Senegal$Diawara	1998	JN995270.1	mosquito	Small
S298ARMcxMRGI2003	Mauritania$Guimi	2003	JN995289.1	mosquito	Small
S303ARMcxMRGI2003	Mauritania$Guimi	2003	JN995290.1	mosquito	Small
S30ARMcxMRAA1999	Mauritania$AyounElAtrouss	1999	JN995275.1	mosquito	Small
S31ARBkcxSNBA2002	Senegal$Barkedji	Jun-05	JN995277.1	mosquito	Small
S32ARBkcxSNBA2002	Senegal$Barkedji	2002	JN995278.1	mosquito	Small
S33ARBkcxSNBA2002	Senegal$Barkedji	Jun-05	JN995279.1	mosquito	Small
S347ARMcxMRGI2003	Mauritania$Guimi	2003	JN995291.1	mosquito	Small
S356ARMcxMRGI2003	Mauritania$Guimi	2003	JN995292.1	mosquito	Small
S35ARBkcxSNBA2003	Senegal$Barkedji	2003	JN995293.1	mosquito	Small
S36ARBkcxSNBA2003	Senegal$Barkedji	2003	JN995294.1	mosquito	Small
S9ARDwcxSNDI1998	Senegal$Diawara	1998	JN995271.1	mosquito	Small
Zinga	CentralAfricanRepublic	1969	DQ380167.1	mosquito	Small
ZM657	Egypt$Sharqiya	31-May-05	DQ380146.1	mosquito	Small
169508	Madagascar	Apr-08	GQ443167.1	ovine	Small
2007000473	Kenya$Kajaidodistrict	26-Jan-07	EU574080.1	ovine	Small
2007003644	Kenya$Baringodistrict	7-Feb-07	EU574059.1	ovine	Small
Kenya57Rintoul	Kenya	1951	DQ380155.1	ovine	Small
SA51VanWyck	SouthAfrica$Boshof	1951	DQ380158.1	ovine	Small
35/74	SouthAfrica	1974	JF784388.1	sheep	Small
Kakamas	SouthAfrica	Oct-09	JQ068142.1	sheep	Small
S11ANMMRHG1998	Mauritania$HodhElGarbi	1998	JN995264.1	sheep	Small
S18ANBkSNBA1993	Senegal$Barkedji	1993	JN995261.1	sheep	Small
S24ANMMRHG1998	Mauritania$HodhElGarbi	1998	JN995267.1	sheep	Small
S25ANMMRHG1998	Mauritania$HodhElGarbi	1998	JN995268.1	sheep	Small
ZS6365	Egypt$Gharbiya	1-Jun-05	DQ380145.1	sheep	Small
KEN/Bar032/07	Kenya	3-Feb-07	HM586979.1	Unknown	Small
KEN/Bar035/07	Kenya	30-Jan-07	HM586980.1	Unknown	Small
KEN/Gar004/06	Kenya	21-Dec-06	HM586975.1	Unknown	Small
KEN/Gar008/06	Kenya	21-Dec-06	HM586976.1	Unknown	Small
KEN/Kil006/07	Kenya	5-Jan-07	HM586977.1	Unknown	Small
KEN/Mal032/07	Kenya	15-Jan-07	HM586978.1	Unknown	Small
MP12	Egypt	1977	DQ380154.1	Unknown	Small
S16ANMMRHG1998	Mauritania$HodhElGarbi	1998	JN995265.1	Unknown	Small
S22ANMMRHG1998	Mauritania$HodhElGarbi	1998	JN995266.1	Unknown	Small
Smithburn	Uganda	1944	DQ380157.1	Unknown	Small
TAN/Dod002/07	Tanzania	14-Mar-07	HM586982.1	Unknown	Small
TAN/Tan001/07	Tanzania	8-Feb-07	HM586981.1	Unknown	Small
